# Nanoscale flow cytometry‐based quantification of blood‐based extracellular vesicle biomarkers distinguishes MCI and Alzheimer's disease

**DOI:** 10.1002/alz.14087

**Published:** 2024-07-03

**Authors:** Thamara Dayarathna, Austyn D. Roseborough, Janice Gomes, Reza Khazaee, Carolina R. A. Silveira, Kathy Borron, Soojung Yu, Kristy Coleman, Sarah Jesso, Elizabeth Finger, Penny MacDonald, Michael Borrie, Jennie Wells, Robert Bartha, Guangyong Zou, Shawn N. Whitehead, Hon S. Leong, Stephen H. Pasternak

**Affiliations:** ^1^ Institute for Genomic Medicine Abigail Wexner Research Institute at Nationwide Children's Hospital Columbus Ohio USA; ^2^ Vulnerable Brain Lab, Department of Anatomy and Cell Biology, Schulich School of Medicine and Dentistry Western University London Ontario Canada; ^3^ Robarts Research Institute, Schulich School of Medicine and Dentistry Western University London Ontario Canada; ^4^ Department of Biology Western University London Ontario Canada; ^5^ Biotron Integrated Microscopy Facility Western University London Ontario Canada; ^6^ Cognitive Neurology and Alzheimer's Disease Research Centre Parkwood Institute, St. Joseph's Health Care Centre London Ontario Canada; ^7^ Department of Clinical Neurological Sciences, Schulich School of Medicine and Dentistry Western University London Ontario Canada; ^8^ Department of Geriatric Medicine, Schulich School of Medicine and Dentistry Western University London Ontario Canada; ^9^ Department of Epidemiology and Biostatistics, Schulich School of Medicine and Dentistry Western University London Ontario Canada; ^10^ Sunnybrook Research Institute University of Toronto Toronto Ontario Canada

**Keywords:** Alzheimer's disease, biomarker, dementia, extracellular vesicle, mild cognitive impairment, nanoflow cytometry

## Abstract

**INTRODUCTION:**

Accurate testing for Alzheimer's disease (AD) represents a crucial step for therapeutic advancement. Currently, tests are expensive and require invasive sampling or radiation exposure.

**METHODS:**

We developed a nanoscale flow cytometry (nFC)‐based assay of extracellular vesicles (EVs) to screen biomarkers in plasma from mild cognitive impairment (MCI), AD, or controls.

**RESULTS:**

Circulating amyloid beta (Aβ), tau, phosphorylated tau (p‐tau)181, p‐tau231, p‐tau217, p‐tauS235, ubiquitin, and lysosomal‐associated membrane protein 1–positive EVs distinguished AD samples. p‐tau181, p‐tau217, p‐tauS235, and ubiquitin‐positive EVs distinguished MCI samples. The most sensitive marker for AD distinction was p‐tau231, with an area under the receiver operating characteristic curve (AUC) of 0.96 (sensitivity 0.95/specificity 1.0) improving to an AUC of 0.989 when combined with p‐tauS235.

**DISCUSSION:**

This nFC‐based assay accurately distinguishes MCI and AD plasma without EV isolation, offering a rapid approach requiring minute sample volumes. Incorporating nFC‐based measurements in larger populations and comparison to “gold standard” biomarkers is an exciting next step for developing AD diagnostic tools.

**HIGHLIGHTS:**

- Extracellular vesicles represent promising biomarkers of Alzheimer's disease (AD) that can be measured in the peripheral circulation.
- This study demonstrates the utility of nanoscale flow cytometry for the measurement of circulating extracellular vesicles (EVs) in AD blood samples.
- Multiple markers including amyloid beta, tau, phosphorylated tau (p‐tau)181, p‐tau231, p‐tau217, and p‐tauS235 accurately distinguished AD samples from healthy controls.
- Future studies should expand blood and cerebrospinal fluid–based EV biomarker development using nanoflow cytometry approaches.

## 1 INTRODUCTION

Neurodegenerative diseases represent a major cost to our health‐care system. In the United States, Alzheimer's disease (AD) affects 6.2 million individuals and without disease‐modifying therapeutics, this number could reach 13.8 million by 2060.^1^ While there are currently no treatments that reverse AD, recent clinical trials provide renewed hope that disease progression can be slowed.^2^, ^3^, ^4^ Despite this progress, a persistent limitation in the research and treatment of AD is the lack of specific and sensitive biomarkers that can detect presymptomatic AD and identify at‐risk individuals. Because it is widely accepted that neurodegenerative disease begins decades before the clinical presentation of AD, early detection is crucial for successful intervention.

Limitations in clinical trials of AD therapeutics may result from treatments being introduced too late in the disease process.^5^, ^6^, ^7^, ^8^ Without reliable biomarkers of early AD, clinical trials are at risk of inaccurate and delayed enrollment of patients.^9^ In addition, the lack of quantitative markers of disease progression means that trials rely primarily on cognitive and clinical testing.^9^, ^10^, ^11^ As a result, to be well powered, Phase 3 studies in early AD typically must enroll 1000 to 2000 patients, follow them for 1 to 2 years, and cost almost 2 billion dollars.^7^, ^12^, ^13^ Having accurate biomarkers of disease onset and progression would allow for smaller, faster, cheaper clinical trials with objective endpoints.

A definitive diagnosis of AD is made by a neuropathologist when examining accumulations of amyloid beta (Aβ) and phosphorylated tau isoforms in a *post mortem* brain. Ideal biomarker approaches would allow for the quantification of these pathological proteins without an autopsy. Although the field of biomarker development is promising, the best‐validated biomarkers rely on invasive cerebrospinal fluid (CSF) sampling, require large radiochemistry laboratories and radiation exposure to the patient (positron emission tomography [PET] scanning) or specialized imaging protocols (magnetic resonance imaging [MRI]). Currently, there are no blood‐based biomarkers for neurodegenerative diseases that are being consistently implemented clinically. The most promising fluid‐based biomarker studies measure levels of Aβ or phosphorylated tau (p‐tau) isoforms in plasma or CSF.^14^, ^15^, ^16^, ^17^, ^18^, ^19^, ^20^, ^21^, ^22^, ^23^ Neurofilament light chain is another promising biomarker for neurodegenerative disease but lacks specificity to AD.^24^, ^25^ Furthermore, many studies remain limited by significant overlap between patients and controls,^26^, ^27^ and the use of assay platforms that are not commonly available.^14^, ^28^, ^29^, ^30^

Extracellular vesicles (EVs) may be promising biomarkers of central nervous system (CNS) diseases.^31^, ^32^, ^33^ EVs are 100 to 1000 nm particles including microvesicles, exosomes, and apoptotic bodies.^34^ EVs bear membrane proteins and cargo specific to their cell of origin, and have been implicated in many aspects of neurodegenerative disease.^35^, ^36^, ^37^ Importantly, EVs cross the blood–brain barrier (BBB) by transcytosis or exit the CNS via CSF flow through arachnoid granulations, making them readily detectable within bodily fluids.^37^ Several studies have targeted brain‐derived EVs in blood using antibodies against neural‐specific adhesion molecules (e.g., neural cell adhesion molecule [NCAM], L1 cell adhesion molecule [L1‐CAM]).^38^, ^39^ Using these approaches, neuron‐derived EVs from AD patients demonstrated increased levels of p‐tau181 and Aβ42 compared to controls.^38^, ^40^ In patients with mild cognitive impairment (MCI), elevated levels of p‐tau181, p‐tauS396, and Aβ42 and lower levels of neurogranin predict progression to AD.^41^ EVs, therefore, represent an opportunity to non‐invasively measure pathological processes before clinical detection is feasible. However, clinical assessment of EV‐bound proteins is challenged by a lack of consistent and high‐throughput measurement techniques.

While there are many different approaches to the measurement of circulating EVs in blood, they differ in reliability and assay complexity. Nanoscale flow cytometry (nFC) is an emerging technology for EV measurement similar to conventional flow cytometry but optimized for the resolution of 80 to 1000 nm particles. This technology provides numerous advantages over standard biochemical techniques. nFC uses tiny volumes of patient plasma (10–40 µL), requires minimal sample handling, and has capacity to analyze multiple biomarkers simultaneously. It provides rapid readouts, examining tens of thousands of EVs per second, and is directly quantitative. The primary objective of this study was to develop nFC‐based quantification of brain‐derived EV biomarkers that can discriminate MCI and AD. Working with plasma samples from a cohort of clinically diagnosed MCI and AD patients from our clinic and cognitively normal controls, > 30 candidate targets were screened including Aβ, oligomeric amyloid, fibrillar amyloid, various p‐tau isoforms, ubiquitin, alpha‐synuclein, and lysosomal‐associated membrane protein 1 (LAMP1). We report successful discrimination of MCI and AD plasma samples using a novel and rapid nFC‐based approach with multiple individual biomarkers and biomarker combinations achieving area under the receiver operating characteristic curve (AUC) values > 0.96 and sensitivity/specificity > 0.96.

## 2 METHODS

### 2.1 Patient selection

Patient groups were recruited from the Cognitive Neurology and Alzheimer Research Centre (CNARC) at Parkwood Institute, St. Joseph's Health Care Centre in London, Ontario, Canada by neurologists specialized in the diagnosis of neurodegenerative disease (Drs. Pasternak and Finger) with clinical trial experience. CNARC is a center for the Ontario Brain Institute's Ontario Neurodegenerative Disease Research Initiative (ONDRI study), the Canadian Consortium on Neurodegeneration in Aging (CCNA; The COMPASS‐ND study), and the National Institute of Health's Alzheimer's Disease Neuroimaging Initiative (ADNI).  Normal controls were drawn from unaffected family members, caregivers, and volunteers attending cognitive research studies and were screened for normal cognition. Inclusion criteria were patients who were diagnosed through normal clinic operations including a history and physical, basic blood work, and neuroimaging and based upon published clinical diagnostic criteria for MCI^42^, ^43^ and AD.^44^, ^45^ Exclusion criteria were (1) clinical, imaging, or laboratory evidence of another disease causing neurological symptoms (e.g., significant head injury or stroke, substance abuse) or (2) unstable psychiatric disease (depression is acceptable if treated).

**RESEARCH IN CONTEXT**

1. **Systematic review**: Rapid and accurate diagnostic testing for Alzheimer's disease (AD) represents a crucial step for the advancement of therapeutics and disease management. Current tests for AD are expensive, require specialized sample handling, invasive sampling methods, or radiation exposure. Recently, there has been much interest in the use of extracellular vesicles (EVs) as diagnostic indicators of AD.
2. **Interpretation**: Findings of this study detail the use of nanoscale flow cytometry for the measurement of circulating EVs in AD blood samples. Multiple markers including amyloid beta, tau, phosphorylated tau (p‐tau)181, p‐tau231, p‐tau217, and p‐tauS235 accurately distinguished AD samples from healthy controls.
3. **Future directions**: Incorporating nanoscale flow cytometry–based measurements of EVs for the utility of AD biomarkers in larger patient populations and comparison to “gold standard” biomarkers (amyloid and tau levels in cerebrospinal fluid, positron emission tomography imaging) is an exciting next step in the development of clinical diagnostic tools for AD.

### 2.2 Cognitive assessment

Patients and controls completed our routine screening battery that includes MoCA (Montreal Cognitive Assessment), MMSE (Mini‐Mental State Examination), Word Fluency, Clock Draw, and Trail Making Test Parts A and B (TMT A/B). Samples were also collected from healthy control (HC) subjects who are cognitively normal (MoCA > 26; *n* = 33) and typically are family members or caregivers of patients seen in our clinics. Patients were stratified into groups with MCI (*n* = 17), mild AD (*n* = 33; MMSE > 20), moderate AD (*n* = 15; MMSE 10–20), and severe AD (*n* = 11; MMSE < 10).

### 2.3 Plasma collection

Blood was collected after venipuncture into ethylenediaminetetraacetic acid–coated collection tubes and centrifuged at 1000 g  for 10 minutes at 4°C. Isolated plasma was stored at −80°C until the time of analysis.

### 2.4 Marker selection

A list of putative biomarker candidates for nFC was selected based on previously published studies of AD plasma biomarkers, previous studies of brain‐derived EVs, and additional markers of interest involved in AD pathophysiology. p‐tau181,^18^, ^21^ p‐tau231,^46^ p‐tauS235,^46^ p‐tau217,^20^ alpha‐synuclein,^47^ beta‐synuclein,^48^ neurofilament,^20^ oligomeric amyloid,^49^ and TAR DNA‐binding protein 43 (TDP‐43)^50^ have been measured in AD plasma or CSF previously. CD171,^41^ CD56,^51^ Aβ42,^41^, ^52^ synaptophysin,^53^ synaptotagmin,^53^ neurogranin,^41^ LAMP1,^54^ ubiquitin,^55^ p‐tauS396^52^ have been reported on brain‐derived EVs. Additional tau phosphoisoforms including p‐tauS202/T205, T18, S199, S262, and S422 were included as they are increased in AD but have not been evaluated as biomarkers.^56^

### 2.5 Nanoscale flow cytometry

Antibodies were purchased preconjugated or covalently conjugated with fluorescent dyes using commercially available kits when conjugated versions were not available. Antibodies included p‐tau181 (BioLegend, 846608), p‐tau231 (Invitrogen, 44‐746G), p‐tauS235 (PA5‐104785), p‐tauS202‐T205 (Invitrogen, MN1020), p‐tau217 (Invitrogen, 44‐744), ubiquitin (Sigma, SAB1306582), LAMP1 (Sigma, L1418), synaptophysin (Biorbyt, orb485757), oligomeric amyloid (abcam, ab183460), fibrillar amyloid (EMD Millipore, MABN687), neurofilament light chain (Novus Biologicals, NBP2‐47970F), TDP‐43 (Protein Tech, 10782‐2‐AP). Antibody labeling kits (AF‐488 Invitrogen A20181, AF‐647 Invitrogen A20186, 405 NGS‐ester 1861106 Thermo Scientific) were used according to kit directions. Plasma samples (10 µL) were incubated in triplicate with antibody for 30 minutes at room temperature and diluted up to 1000 µL of phosphate‐buffered saline (PBS) prior to analysis on the Apogee A50 Microplus Nanoflow Cytometer (Apogee Flow Systems Inc.). Instrument settings to achieve data linearity using the Apogee cytometer have been previously published and followed in this study.^57^ Datagrams of unlabeled buffer and plasma are presented in Figure S1 in supporting information. Prior to running plasma samples, it was ensured that background levels of buffer (PBS) did not exceed 100 EVs/s and standardized beads were run weekly to confirm they were measured at their stock concentration of 5000 EVs/s. Instrument settings included sheath pressure: 150 mbar, flow rate: 150 µL/min for 130 µL, lasers: 100 mW 405 nm (violet), 70 mW 638 nm (red), 70 mW 488 (green). Light scatter of events was produced using the 405 nm laser, with thresholds to eliminate background noise of 34 a.u. for small angle light scatter (SALS) and 21 a.u. for long angle light scatter (LALS). Photomultiplier tube (PMT) voltages: LALS (265 V), SALS (340 V), L488‐Grn (525 V), L638‐Red (650). Figure S2 in supporting information includes polystyrene and silicon sizing beads for comparison of EV sizes. nFC of dilution reagent (PBS), single antibodies, and unlabeled plasma are reported in Figure S2. Outputs of EV numbers are reported as events/µL, which represents the concentration of labeled particles detected after gating by fluorescent channel of antibodies used.

### 2.6 EV isolation

Prior to enzyme‐linked immunosorbent assay (ELISA) and western blot EVs were isolated via ultracentrifugation, immunoprecipitation, or size exclusion chromatography (SEC) depending on desired preparation for subsequent experiments. Fir ultracentrifugation, plasma was centrifuged at 15,000 g for 15 minutes at 4°C to remove cellular debris. Fifty mL of 10 patient plasma samples from each group were combined and concentrated using Amicon filter (100kDA size cutoff). Concentrated media was ultracentrifuged at 100,000 g for 1 hour and 15 minutes at 4°C prior to resuspension in 50 µL PBS. For SEC, circulating EVs in patient plasma were fractionated using Bio‐Gel A 1.5 m agarose beads (Bio‐Rad, cat# 151‐0450). In this protocol, 2 mL of gel was loaded onto Poly–Prep® Chromatography Columns (Bio‐Rad Cat# 7311550), and the beads were allowed to settle under gravity. After settling, beads in the column were washed two times with 2 mL of 1× PBS. The final PBS wash was allowed to run through the column completely under gravity. Then, 50 µL of plasma was loaded onto the column and allowed to completely flow into beads. One mL of PBS was loaded carefully to the column and 100  µL of flow‐through fractions were collected. For immunoprecipitation, Pierce Protein G agarose beads (20397; 50 µL slurry) were washed twice with 1× PBS (500 µL each). Beta‐III tubulin (ab78078‐100; 10 µL) was incubated with the washed protein G agarose beads with end‐end rotation for 1 hour at room temperature and plasma sample (50 µL) was added to the bead antibody mixture and incubated overnight at 4°C with end–end rotation. The next day, the sample was centrifuged at 500 g for 5 minutes. Supernatant was removed and beads were washed twice with PBS. Bead sample was fixed with freshly prepared glutaraldehyde solution and submitted for electron microscopy analysis.

### 2.7 Western blot

Protein assays of EVs isolated from plasma samples were performed using bicinchoninic acid assays (Pierce). For western blotting, 5 µg of protein per lane was loaded into a 4% to 12% gradient gel and separated via gel electrophoresis prior to transfer onto nitrocellulose membranes. Membranes were blocked with milk and probed with antibodies against CD9 (Invitrogen TS9 106261), LAMP1 (Sigma, L1418), tau (abcam, ab254256), alpha‐synuclein (Santa Cruz, sc‐12767), and oligomeric amyloid (abcam, ab126892) prior to visualization using chemiluminescent enzyme substrate.

### 2.8 ELISA

Using ELISA kits, concentrations of alpha‐synuclein a, ab260052), tau (Invitrogen, KHB004) and Aβ42 (Invitrogen, KHB3544) in neat plasma were measured according to manufacturer's instructions.

### 2.9 Transmission electron microscopy

Purified EV samples were fixed with 2% paraformaldehyde and 0.1% glutaraldehyde in 0.1 M phosphate buffer (pH 7.0) for 1 hour at 4°C. Five to seven µL of fixed EV samples were loaded on carbon‐coated grids (CF300‐Ni 215‐412‐8400) and were incubated for 5 minutes at 4°C. The grids were blotted from the side with filter paper and were rinsed with 100 µL of 1× PBS three times each for 5 minutes. Then, the grids were briefly touched with a drop of Methylamine Tungstate Negative Stain (ThermoFisher, A51036). The grids were incubated with second drops of stain for 1 minute. The grids were incubated with 50 µL of 0.05 M glycine for 10 minutes to quench free aldehyde groups. Then, the grids were transferred to a drop of blocking solution (1% bovine serum albumin [BSA] in 1× PBS) for 30 minutes. Afterward, the grids were incubated with 50 µL of the primary antibodies (Aβ42 [custom in‐house antibody], 1:50; p‐tauS235 [PA5104785]), 1:30; p‐tau217 [MM‐0148‐P], 1:100) for 1 hour at room temperature. Grids were washed with five drops of 1× PBS containing 0.1% BSA for 10 minutes each. Then, the grids were incubated with a drop of secondary antibody for 1 hour at room temperature. Depending on the primary antibodies, the secondary antibodies were either goat antimouse conjugated to 6 nm gold particles (Aurion GAM‐GG10518) or goat antirabbit conjugated to 10 nm gold particles (Aurion GAR‐10714/1; diluted in PBS containing 0.1% BSA). Grids were washed with five drops of 1x PBS containing 0.1% BSA (Aurion 10212/1V) for 10 minutes each and then were washed with ddH_2_O two times prior to visualization. Imaging of isolated EVs was carried out using a transmission electron microscope CM10 (Philips Electron Optics).

### 2.10 Statistical analyses

Group comparisons were performed on Prism 9 (GraphPad) using non‐parametric Kruskal–Wallis test with a Dunn post hoc test for multiple comparisons. The overall diagnostic value of a biomarker was quantified using the AUC, estimated by a non‐parametric method as implemented in R package pROC on RStudio.^58^, ^59^ For multiple markers: markers were combined either by summation or using logistic regression to determine individual coefficients for each marker. Results were presented for point estimates of AUC and associated 95% confidence intervals (CIs) as well as the values for sensitivity and specificity at the optimal threshold, which results in maximum sum of sensitivity and specificity.

## 3 RESULTS

A total of 104 plasma samples were collected from HCs (*n* = 26), subjects with MCI (*n* = 23), subjects with mild AD (*n* = 32), moderate AD (*n* = 15), and severe AD (*n* = 10). All participants were recruited from memory clinics at Parkwood Institute in London, Canada. Demographics of the selection patient population are reported in Table 1. HC samples were significantly younger and had better performance on MMSE, MoCA, and TMT A/B than the MCI and AD diagnostic groups (Table 1).

**TABLE 1:** Demographics of selected patient population including sex, age, and cognitive testing results.

			AD		
	HC (*N* = 26)	MCI (*N* = 23)	Mild (*N* = 32)	Moderate (*N* = 15)	Severe (*N* = 10)
Sex (M/F)	12/14	10/13	14/18	7/8	6/4
Age (years)					
Mean (SD)	63.81 (9.41)	73.82 (8.53)	75.75 (9.2)	71.86 (10.92)	68.4 (11.4)
Range	48–79	56–90	55–92	56–93	44–81
MMSE					
Mean (SD)	29.62 (0.56)	27.13 (1.70)	22.74 (2.18)	15.66 (2.38)	6 (2.7)
Range	28–30	24–30	19–29	11–20	3–11
MoCA					
Mean (SD)	28.42 (1.29)	21.41 (3.77)	15.24 (3.29)	8.25 (2.90)	Inc.
Range	26–30	13–27	8–23	5–15	
Trails A					
Mean (SD)	31.56 (11.07)	44.86 (13.07)	83.35 (39.77)	Inc.	Inc.
Trails B					
Mean (SD)	74 (42.23)	153.90 (68.08)	214.22 (71.81)	Inc.	Inc.

*Notes*: In moderate–severe cases, cognitive testing with MoCA or Trails A/B was often incomplete due to disease severity, marked “Inc.”

Abbreviations: AD, Alzheimer's disease; HC, healthy control; MCI, mild cognitive impairment; MMSE, Mini‐Mental State Examination; MoCA, Montreal Cognitive Assessment; SD, standard deviation; Trails A/B, Trail Making Test Parts A and B.

### 3.1 Distinction of AD samples from HCs using single marker labeling

**FIGURE 1:**
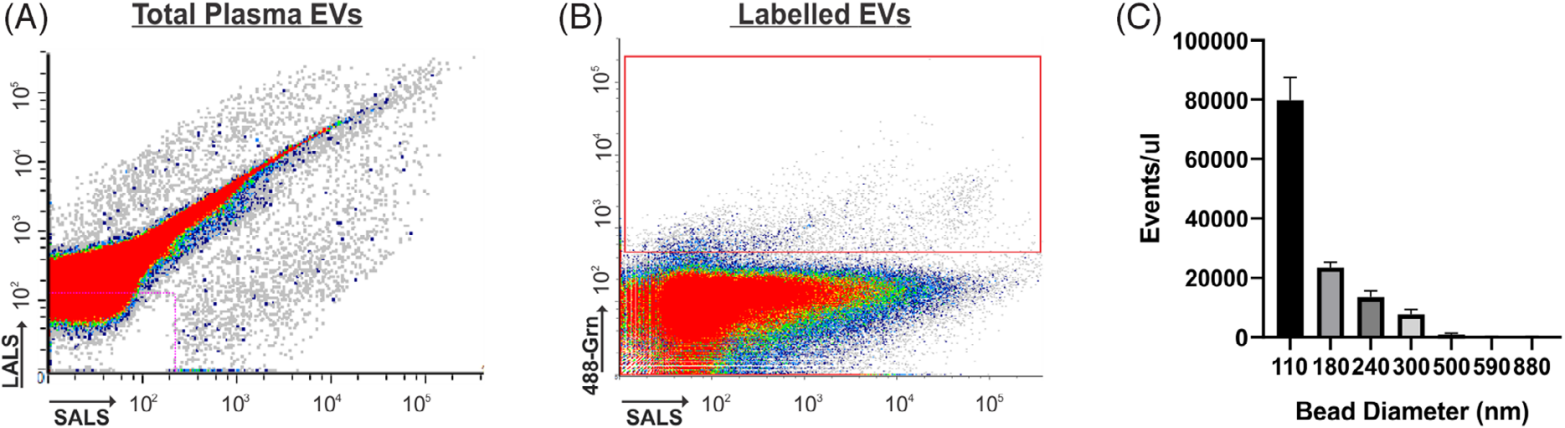
Nanoflow cytometry labeling of plasma EVs. A, Datagram of total plasma EV distribution by light scatter. B, Datagram of EVs labeled with a 488‐conjugated antibody against fibrillar amyloid. Red box indicates ROI of positively labeled particles. C, Particle count according to light scatter of standardized sizing beads (110 and 500 nm silicon beads, 280, 300, 500, 590, and 880 nm polystyrene beads). EVs, extracellular vesicles; LALS, long angle light scatter; ROI, region of interest; SALS, small angle light scatter

**FIGURE 2:**
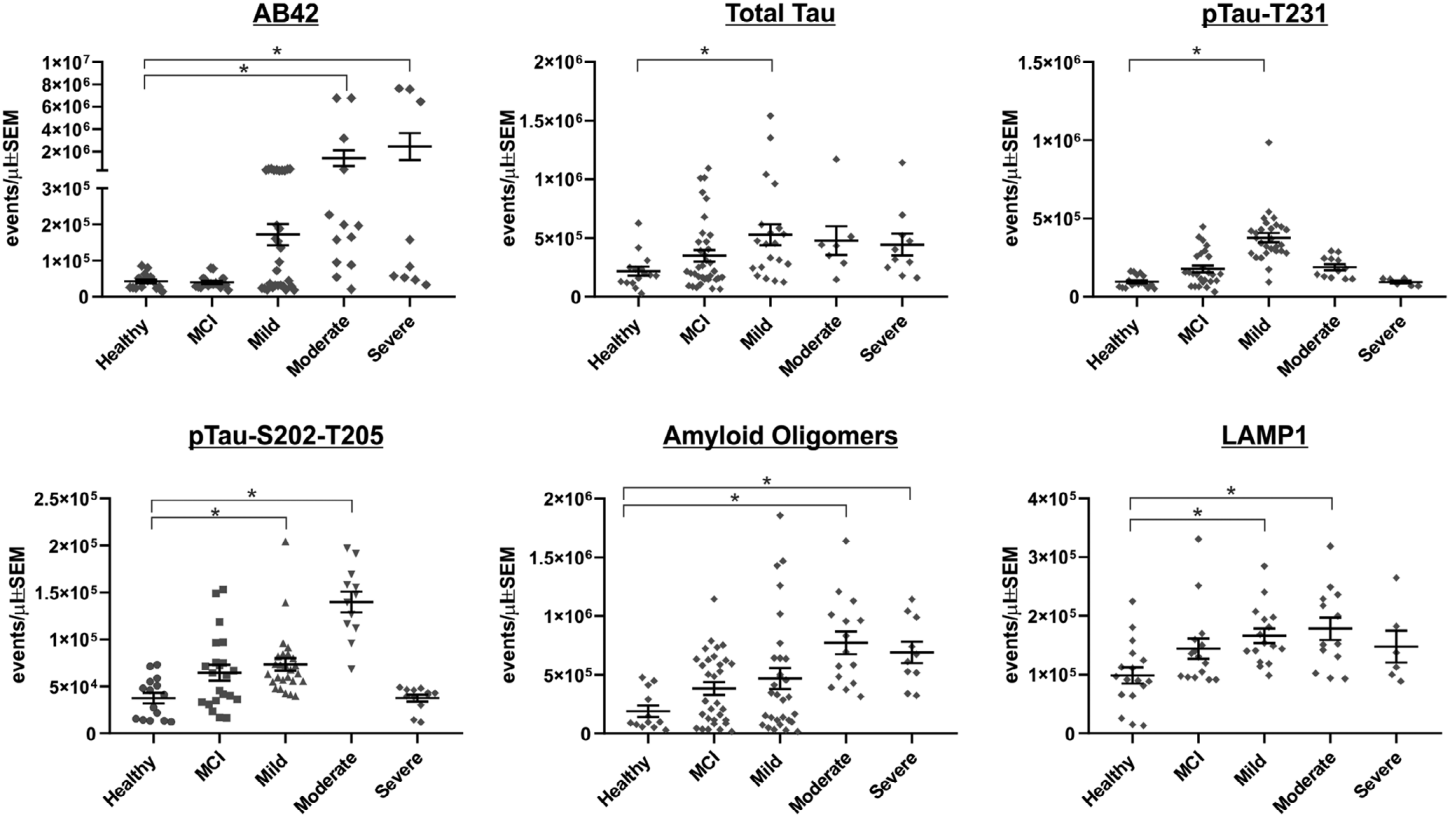
Individual markers that label significantly more EVs in Alzheimer's disease plasma samples compared to controls. Fluorescently labeled antibodies against the indicated proteins were incubated with plasma samples from controls, individuals with MCI or Alzheimer's disease (separated into mild, moderate, and severe categories). Each data point represents the average of triplicate incubations. Statistical comparisons were performed using Prism 9 (GraphPad) with Kruskal–Wallis, Dunn post hoc test for multiple comparisons, and a significance value set at *P *< 0.05. Aβ, amyloid beta; EVs, extracellular vesicles; LAMP1, lysosomal‐associated membrane protein 1; MCI, mild cognitive impairment; ptau, phosphorylated tau; SEM, standard error of the mean

**FIGURE 3:**
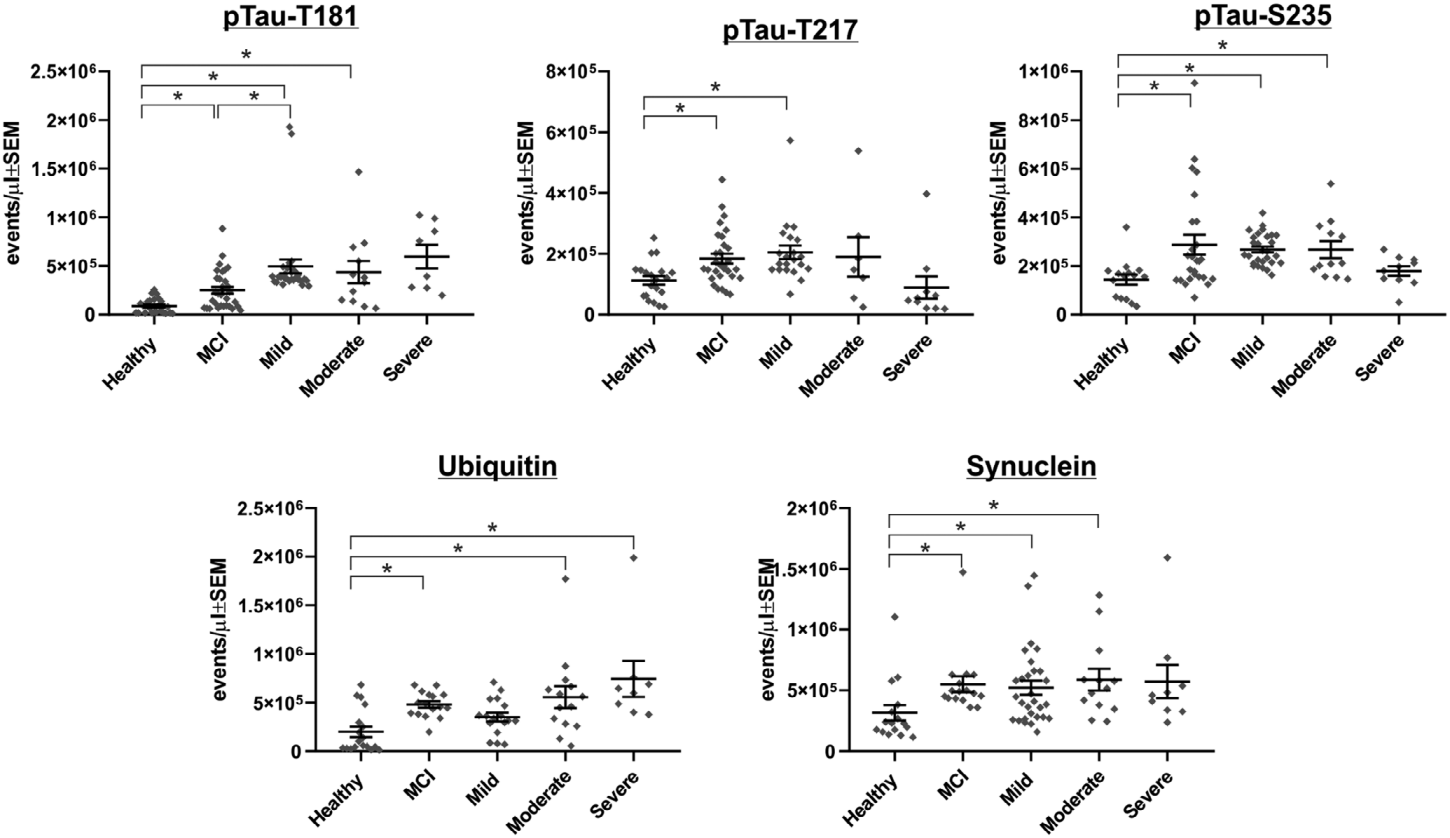
Individual markers that label significantly more EVs in MCI and Alzheimer's disease plasma samples compared to controls. Fluorescently labeled antibodies against the indicated proteins were incubated with plasma samples from controls, individuals with MCI or Alzheimer's disease (separated into mild, moderate, and severe categories). Each data point represents the average of triplicate incubations. Statistical comparisons performed using Prism 9 (GraphPad) with Kruskal–Wallis, Dunn post hoc test for multiple comparisons, and a significance value set at *P *< 0.05. EVs, extracellular vesicles; MCI, mild cognitive impairment; ptau, phosphorylated tau; SEM, standard error of the mean

A list of candidate biomarkers was identified, and each marker was evaluated individually to determine its ability to distinguish diagnostic groups using nFC‐based identification of labeled particles. Datagrams of total plasma EVs, labeled plasma EVs, and size distribution of labeled particles compared to standardized reference beads are provided in Figure 1. Individual antibodies that distinguish AD samples from HC or MCI are displayed in Figures 2 and 3. Compared to HC samples, mild AD samples had significantly elevated levels of events labeled with antibodies against tau (*P* = 0.0108), p‐tau231 (*P* < 0.0001), p‐tau181 (*P* < 0.0001), p‐tau217, p‐tauS235 (*P* = 0.0001), p‐tauS202‐T205 (*P* = 0.0061), alpha‐synuclein (*P* = 0.0200), and LAMP1 (*P* = 0.0046). Moderate AD samples had significantly elevated levels events labeled with antibodies against Aβ42 (*P* = 0.0029), p‐tauS202‐T205 (*P* < 0.0001), p‐tau181 (*P* = 0.0003), p‐tau217 (*P* = 0.0036), p‐tauS235 (*P* = 0.0274), oligomeric amyloid (*P* < 0.0001), alpha‐synuclein (*P* = 0.0082), ubiquitin (*P* = 0.0050), and LAMP1 (*P* = 0.0067). Severe AD had significantly elevated levels of events labeled with antibodies against Aβ42 (*P* = 0.0342), amyloid oligomers (*P* = 0.0004), and ubiquitin (*P* = 0.0003). EVs labeled with antibodies against NCAM, L1‐CAM, synaptophysin, and neurogranin were not significantly different across groups.

### 3.2 Distinction of MCI and AD samples from HCs using single marker labeling

Figure 3 displays antibodies that labeled significantly more particles in MCI plasma samples than those from controls. Compared to HC samples, MCI samples had significantly elevated levels of events labeled with p‐tau181 (*P* = 0.003), p‐tau217 (*P* = 0.0217), p‐tauS235 (*P* = 0.0247), alpha‐synuclein (*P* = 0.0026), and ubiquitin (*P* = 0.0031).

### 3.3 Analysis of receiver operating characteristic curves for individual biomarkers

Receiver operating characteristic (ROC) curves were generated for each individual marker to examine the accuracy of separating AD patients from HC. Antibodies to p‐tau231, p‐tau181, LAMP1, p‐tauS235, oligomeric Aβ, synuclein, ubiquitin, and p‐tau217 all demonstrate AUCs of > 0.75. Of these antibodies, the best single antibody is p‐tau231 with an AUC of 0.96 with sensitivity of 0.85 and a specificity of 1.0 at the optimal threshold. Other promising antibodies include p‐tau181 (AUC of 0.92 with sensitivity 0.91 and specificity 0.93), p‐tauS235 (AUC of 0.88 with sensitivity 0.90 and specificity of 0.86), LAMP1 (AUC of 0.83 with sensitivity of 0.72 and specificity of 0.83), oligomeric Aβ (AUC of 0.81 with sensitivity 1.0 and specificity 0.54), synuclein (AUC of 0.80 with sensitivity 0.67 and specificity 0.91), ubiquitin (AUC of 0.799 with sensitivity of 0.71 and specificity of 0.85), and p‐tau217 (AUC of 0.78 with sensitivity of 0.80 and specificity of 0.63). Table 2 presents the results of ROC analyses along with optimal particle cut‐offs for each individual marker tested and individual curves are presented in Figure S3 in supporting information.

**TABLE 2:** ROC analysis of individual markers.

Antigen	AUC [95% CI]	Cutoff events/µL	Sensitivity	Specificity
p‐tau231	0.957 [0.909, 1.0]	165483	0.850	1
p‐tau181	0.923 [0.818, 1.0]	208250	0.905	0.929
p‐tauS235	0.882 [0.755, 1.0]	182666	0.900	0.857
LAMP1	0.829 [0.705, 0.952]	112441	0.722	0.829
Oligomeric Aβ	0.810 [0.693, 0.926]	480950	1	0.536
Synuclein	0.804 [0.664, 0.945]	241433	0.667	0.909
Ubiquitin	0.799 [0.660, 0.939]	253450	0.706	0.846
p‐tau217	0.782 [0.644, 0.920]	146866	0.800	0.632
Total tau	0.744 [0.605, 0.883]	240100	0.650	0.788
Aβ42	0.743 [0.617, 869]	86716	0.619	0.929
p‐tauS202/T205	0.730 [0.584, 0.876]	28833	0.412	0.960
NfL	0.628 [0.513, 0.831]	306250	0.700	0.694

*Notes*: For each individual marker labeled in plasma, ROC analysis was run to determine distinction of AD samples from HC samples using the pROC package in RStudio.^58^

Abbreviations: Aβ, amyloid beta; AD, Alzheimer's disease; CI, confidence interval; HC, healthy control; NfL, neurofilament light chain; p‐tau, phosphorylated tau; ROC, receiver operating characteristic curve.

### 3.4 Identifying combinations of markers that distinguish AD and MCI from HCs

**FIGURE 4:**
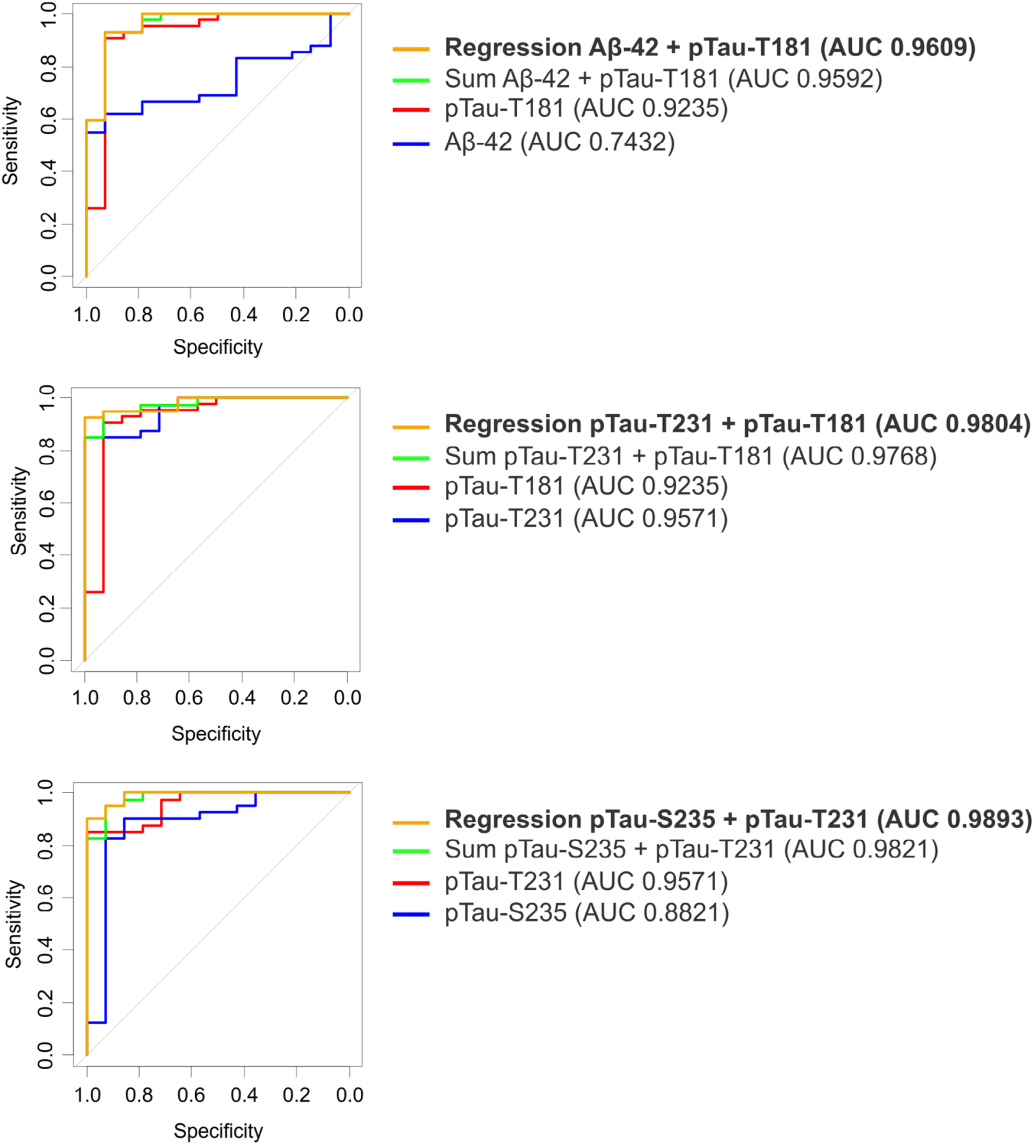
Discrimination of AD samples (mild and moderate) from HC samples using marker combinations. ROC curves of marker combinations using logistic regression to determine coefficients (yellow) or summation (green). ROC curves of individual markers (red and blue). Aβ, amyloid beta; AD, Alzheimer's disease; AUC, area under the receiver operating characteristic curve; HC, healthy control; ptau, phosphorylated tau; ROC, receiver operating characteristic

**FIGURE 5:**
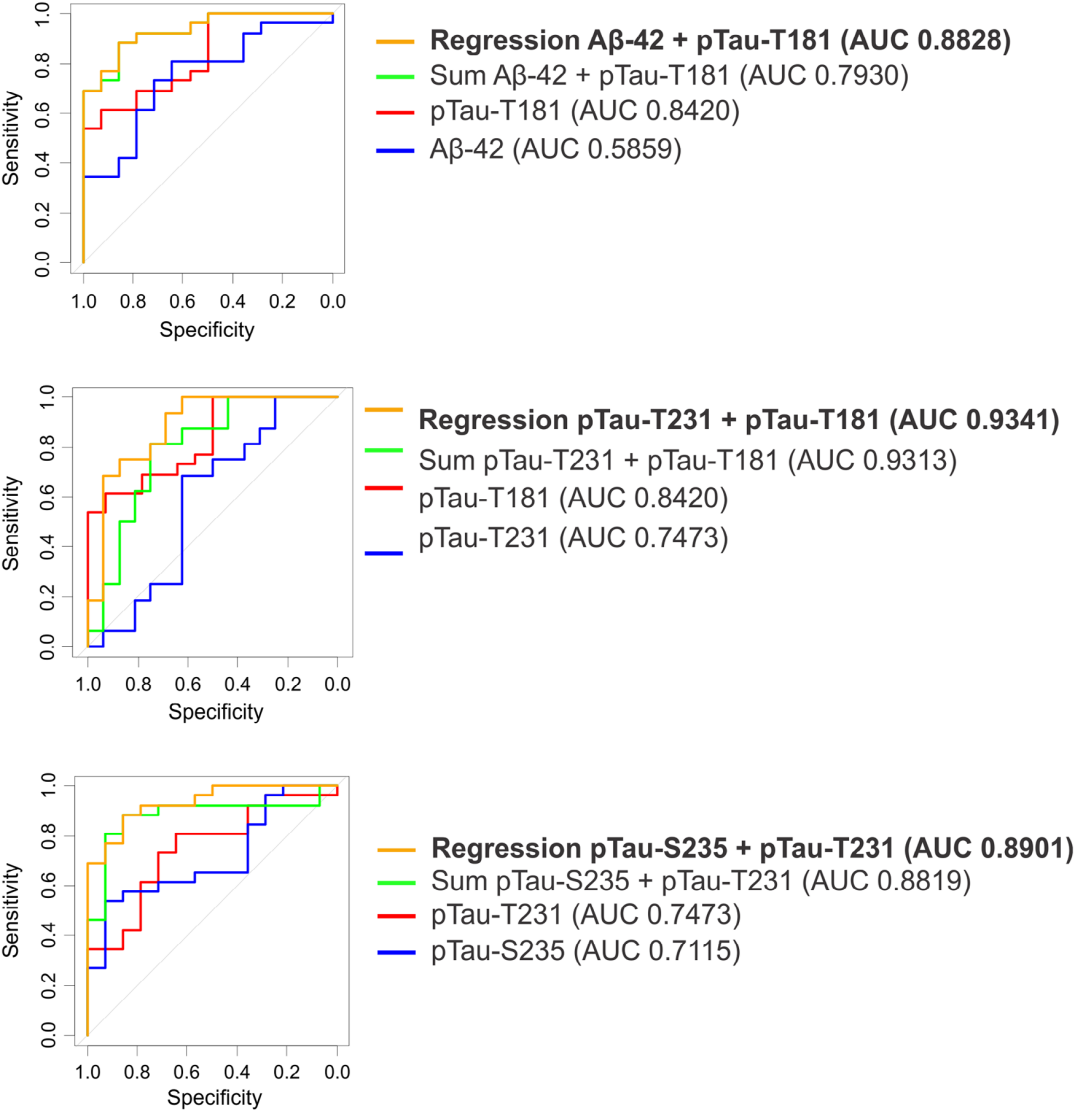
Discrimination of MCI from HC samples using marker combinations. ROC curves of marker combinations using logistic regression to determine coefficients (yellow) or summation (green). ROC curves of individual markers (red and blue). AUC, area under the receiver operating characteristic curve; HC, healthy control; MCI, mild cognitive impairment; ptau, phosphorylated tau; ROC, receiver operating characteristic

As depicted in Figure S4 in supporting information, combinations of markers visually improve discrimination of MCI and AD samples from HC. To quantify the improved discrimination, combinations of biomarkers were evaluated either by summing the markers or using multiple logistic regression to determine individual marker coefficients. Discrimination of AD samples from HC using marker combinations is presented in Figure 4. The combination of p‐tau181 and Aβ42 using either summation (green, AUC 0.96) or multiple logistic regression (orange, AUC 0.96) improves upon the individual AUCs for p‐tau181 (red, AUC 0.92) or Aβ42 (blue, AUC 0.74). The combination of p‐tau181 and p‐tau231 using either summation (green, AUC 0.98) or multiple logistic regression (orange, AUC 0.98) improves upon the individual AUCs for p‐tau181 (red, AUC 0.92) or p‐tau231 (blue, AUC 0.96). The combination of p‐tau231 and p‐tauS235 using either summation (green, AUC 0.98) or multiple logistic regression (orange, AUC 0.99) improves upon the individual AUCs for p‐tau231 (red, AUC 0.96) or p‐tauS235 (blue, AUC 0.88). Discrimination of MCI samples from HC using marker combinations is presented in Figure 5. The combination of p‐tau181 and Aβ42 using either summation (green, AUC 0.79) or multiple logistic regression (orange, AUC 0.88) improves upon the individual AUCs for p‐tau181 (red, AUC 0.84) or Aβ42 (blue, AUC 0.59). The combination of p‐tau181 and p‐tau231 using either summation (green, AUC 0.93) or multiple logistic regression (orange, AUC 0.98) improves upon the individual AUCs for p‐tau181 (red, AUC 0.84) or p‐tau231 (blue, AUC 0.75). The combination of p‐tau231 and p‐tauS235 using either summation (green, AUC 0.88) or multiple logistic regression (orange, AUC 0.89) improves upon the individual AUCs for p‐tauT231 (red,0.75) or p‐tauS235 (blue, AUC 0.71).

### 3.5 Validation and reliability

**FIGURE 6:**
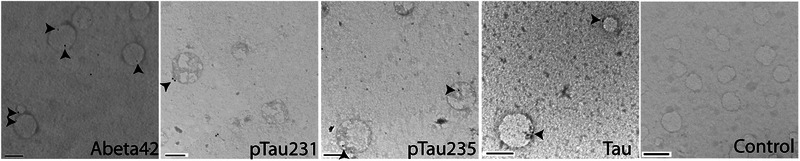
TEM of target markers. Transmission electron microscopy showing immunogold labeling of Aβ, ptau231, ptauS235, and tau on EVs. EVs were isolated from plasma using immunoprecipitation with beta‐III tubulin followed by size exclusion chromatography. Scale bar = 200 nm. Control was generated by omitting the primary antibody. Aβ, amyloid beta; EVs, extracellular vesicles; ptau, phosphorylated tau; TEM, transmission electron microscopy

To determine reliability of nFC assays, we calculated single‐measure intraclass correlations (ICCs) and Cronbach's alpha of Aβ42 and p‐tau181 labeled events in samples after 1 to 4 freeze–thaw cycles. Aβ42 labeled events demonstrated good reliability across freeze–thaw cycles (ICC = 0.82, α = 0.91) and p‐tau181 labeled events demonstrated moderate reliability across freeze–thaw cycles (ICC = 0.7, α = 0.90). This suggests that the management of freeze–thaw cycles is of importance when conducting nFC‐based assays of EV biomarkers. To confirm localization of target proteins, EVs were isolated from pooled plasma samples from each diagnostic group and markers of interest were confirmed via western blot and ELISA (Figure S5 in supporting information). Transmission electron microscopy (TEM) with immunogold labeling was performed on EVs immunoprecipitated out of plasma using a neuron‐specific antibeta III tubulin antibody and subsequently labeled with antibodies against Aβ, tau, p‐tau231, and p‐tauS235. TEM images presented in Figure 6 demonstrate that Aβ42, p‐tau231, p‐tauS235, and tau are all present the outer limiting membrane of EVs.

## 4 DISCUSSION

Therapeutic approaches that slow disease progression will require biomarkers that detect the earliest onset of the disease. In this cross‐sectional study, we report the use of nFC to non‐invasively measure AD biomarkers in an effort to detect MCI patients who would most benefit from earlier treatment. Of the markers tested in this study Aβ, oligomeric amyloid, total tau, p‐tau181, p‐tau231, p‐tau217, p‐tauS235, p‐tauS202‐T205, LAMP1, ubiquitin, and alpha‐synuclein all accurately distinguished MCI and/or AD diagnostic categories. nFC enabled direct quantification of EV‐bound biomarkers in small plasma sample volumes (10 µL) without the need for EV isolation steps. Combinations of markers quantified by nFC provided the most accurate discrimination of diagnostic categories. This can be done simply with summation or using regression analyses to determine coefficients for weighting of individual biomarkers. Either method improves upon the discrimination offered by a single marker alone. The most promising marker combination of p‐tau231 and p‐tauS235 offered excellent distinction of AD samples with an AUC of 0.989.

Of the markers tested, we hypothesized that p‐tau181 would discriminate AD samples given its prevalent examination in prior studies. Excitingly, nFC‐based measurement of p‐tau181 provided better discrimination of AD samples than what has been previously reported with CSF‐based measurement, achieving an AUC of 0.93 with a sensitivity of 0.93 and a specificity of 0.92 in our cohort. A recent Cochrane meta‐analysis suggested that CSF p‐tau181 had a median specificity of 47.5% and sensitivity of 81% for predicting conversion to AD.^60^ CSF p‐tau/Aβ ratio in the studies examined showed sensitivities were between 80% and 96% and specificities between 33% and 95%.^60^ nFC‐based measurement of p‐tau181 provides a much less invasive and more specific method for measuring p‐tau181 than CSF‐based measurements that require lumbar puncture. This blood‐based platform is a promising alternative over CSF‐based analytes.

Additional p‐tau isoforms are being increasingly measured in blood samples via ELISA assays. p‐tau217 has been presented in a number of recent publications as a species of tau that may outperform p‐tau181 in CSF tests for AD.^29^ In this cohort of samples measured with nFC, p‐tau217 did achieve an AUC of 0.78 indicating moderate discriminate capability, but it did not outperform p‐tau181. In fact, p‐tau231 performed the best of all individually measured p‐tau isoforms with an AUC of 0.96. This is promising given recent work suggesting that elevation of p‐tau231 occurs in the earliest stages of Aβ accumulation during preclinical AD.^61^ Surprisingly, p‐tau231 and p‐tauS202/T205 labeled more events in mild or moderate AD than in severe AD plasma. This was unexpected as accumulation of p‐tau accompanies the progression of AD.^56^ There are a few possible explanations for these results: certain p‐tau isoforms may peak at different severities of disease, the release of tau in EVs may change over the course of disease, and the loss of viable neurons may reduce processing of tau into EVs with a subsequent increase in intraneuronal and/or non–EV‐bound extracellular tau.

In addition to the more conventionally measured AD biomarkers, markers that may be indicative of increased proteolytic and lysosomal activity occurring alongside Aβ and tau proteinopathies were also measured in plasma samples. nFC measurement of LAMP1 and ubiquitin‐positive EVs revealed elevations in AD samples. Although both markers are not specific to AD pathology, they may provide information regarding the biological mechanisms of brain‐derived EV formation and release. Ubiquitinylation is thought to be a mechanism leading to shuttling of proteins to small EVs;^62^, ^63^ meanwhile, LAMP1 suggests that EVs are derived from an endosomal–lysosomal origin as opposed to plasma‐membrane–derived vesicles.^64^ Using markers such as LAMP1 and ubiquitin in combination with disease‐specific proteins may be helpful in refining subpopulations of EVs in the future.

A proportion of antibodies that we examined did not distinguish control plasma from AD plasma, which is listed in Table S1 in supporting information and Figure S1. The antigens synaptotagmin, neurogranin, and synaptophysin have been reported to label higher numbers of EVs in AD patients than in controls.^38^ nFC quantification did not replicate these trends. Similarly, antibodies against NCAM and L1‐CAM, which are commonly used to isolate EVs of neuronal origin, did not demonstrate any differences across groups. This may indicate that the total number of neuronal EVs detected by nFC is unchanged although their content differs based on disease state. Many previously published studies of neuronal EVs and their cargo have been analyzed using immunoprecipitation‐based isolation of EVs followed by western blot or ELISA, so it is possible that they are not comparable to nFC‐based labeling of EVs; for example, antigens may not be exposed in intact EVs or the protein corona on EVs may influence immunoreactivity of antibodies and their binding avidity to EVs. This may also reflect differences in epitopes detected by the various commercially available antibodies. Future testing with enrichment for cell‐specific EV populations or antibodies recognizing different epitopes may help resolve these technical issues.

This is not the first use of nFC for the measurement of EVs in AD plasma.^65^ However, our technique simplifies and streamlines the analysis of EVs by skipping traditional EV preparation techniques and offers unique advantages. Previous approaches require the isolation of EVs, usually via ultracentrifugation or SEC, which introduces significant variability due to sample handling, requires hours of sample preparation, and results in substantial EV loss. Previous studies have also measured EV biomarkers in CSF rather than plasma. Although the biology of EV flow in the brain is not well understood, there may be differences in the ability of EVs to cross endothelial cells into circulation versus movement of EVs across ependymal cells into the CSF. EVs from the brain can enter the blood directly via transcytosis across the endothelial layer, or by traveling first through the glymphatic system, which may be a reason for high numbers of circulating EVs. To our knowledge, direct comparisons of EV levels in CSF versus blood have not been reported and nFC‐based measurement of CSF EVs represents an important future area of study.

With respect to the measurement of soluble AD biomarkers (non‐EV‐bound), studies have measured various p‐tau isoforms or other plasma markers using highly sensitive immunoassays. Unlike these approaches, nFC offers the ability to label multiple markers on the same particle and enables the use of cell‐specific proteins to confirm brain specificity. This may be especially important for the distinction of different neurodegenerative diseases because they frequently have overlap in individual biomarker profiles. Overall, the direct nFC‐based labeling approach used in this study will make EV biomarkers more easily transferable to standard clinical laboratories and result in improved diagnostic yield.

Limitations of this study include the small sample size, absence of longitudinal data, and lack of other biomarkers such as CSF or plasma measurements of Aβ, tau, neurofilament, or neuroimaging biomarkers of AD (amyloid or tau PET imaging). Validation of these results in a larger cohort and comparison to established imaging and biomarker measurements is a crucial next step in the development of a reliable nFC‐based assay. Furthermore, it cannot be confirmed which cell type the labeled vesicles originate from given that co‐labeling with cell‐specific markers was not performed. This represents a useful addition to future studies to determine whether protein aggregates are being carried by EVs from neuronal or glial cell populations. Future study in larger cohorts with additional clinical data and neuroimaging is the obvious next step in validating the utility of this promising and accurate EV‐based diagnostic.

## CONFLICTS OF INTEREST STATEMENT

The authors declare no conflicts of interest. Author disclosures are available in the supporting information.

## CONSENT STATEMENT

All human subjects provided informed consent.

## Supporting information

Supporting information

Supporting information
