# Supplementary material for: Nanoscale flow cytometry‐based quantification of blood‐based extracellular vesicle biomarkers distinguishes MCI and Alzheimer's disease
Source: Alzheimers Dement. 2024 Jul 3;20(9):6094–106. doi: 10.1002/alz.14087 (PMC11497682; doi:10.1002/alz.14087)

**Supplemental Information**

**Figure S1:** nFC of A) silicon standardized sizing beads B) polystyrene standardized sizing beads C) unlabeled plasma D) unlabeled buffer (PBS) in 488 channel E) unlabeled plasma in 488 channel F) unlabeled buffer (PBS) in 647 channel G) unlabeled plasma in 647 channel


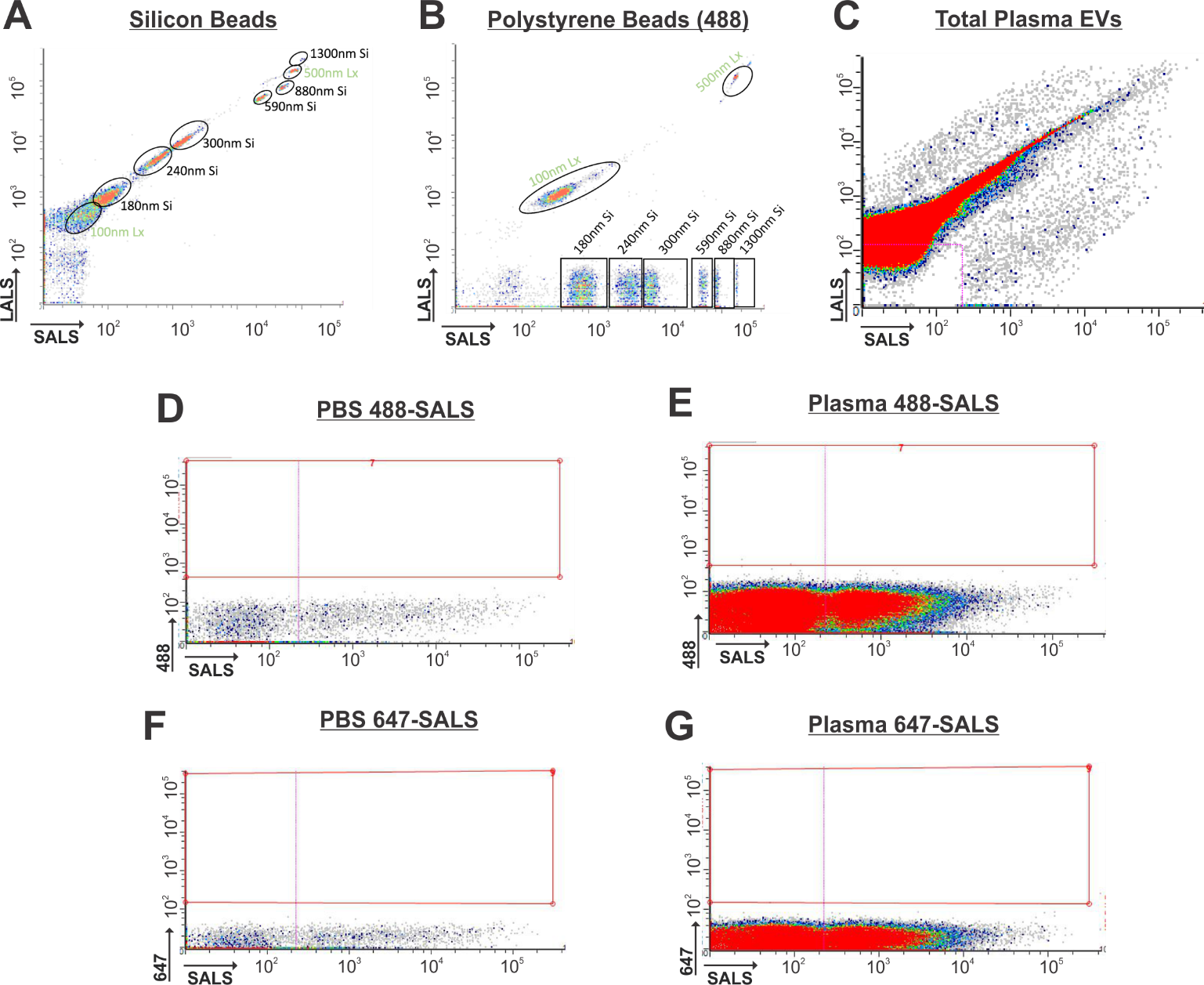


Figure S2: Antibodies that did not distinguish AD or MCI plasma from healthy control samples

**Table S1.** List of Antibodies that do not distinguish Alzheimer’s disease plasma samples from healthy control samples

| CD171 (L1-CAM)  8 tested | CD56 (NCAM)  5 tested | Synaptotagmin | Neurofilament |
| --- | --- | --- | --- |
| Fibrillar beta amyloid | pTau-T22 | Neurogranin | pTau-S262 |
| pTau -S422 | pTau-S199S202 | Synaptophysin | pTau-T18 |
| pTau-S396 | alpha synuclein | Beta-Synuclein | TDP-43 |

**Figure S3:** ROC curves for individual biomarkers


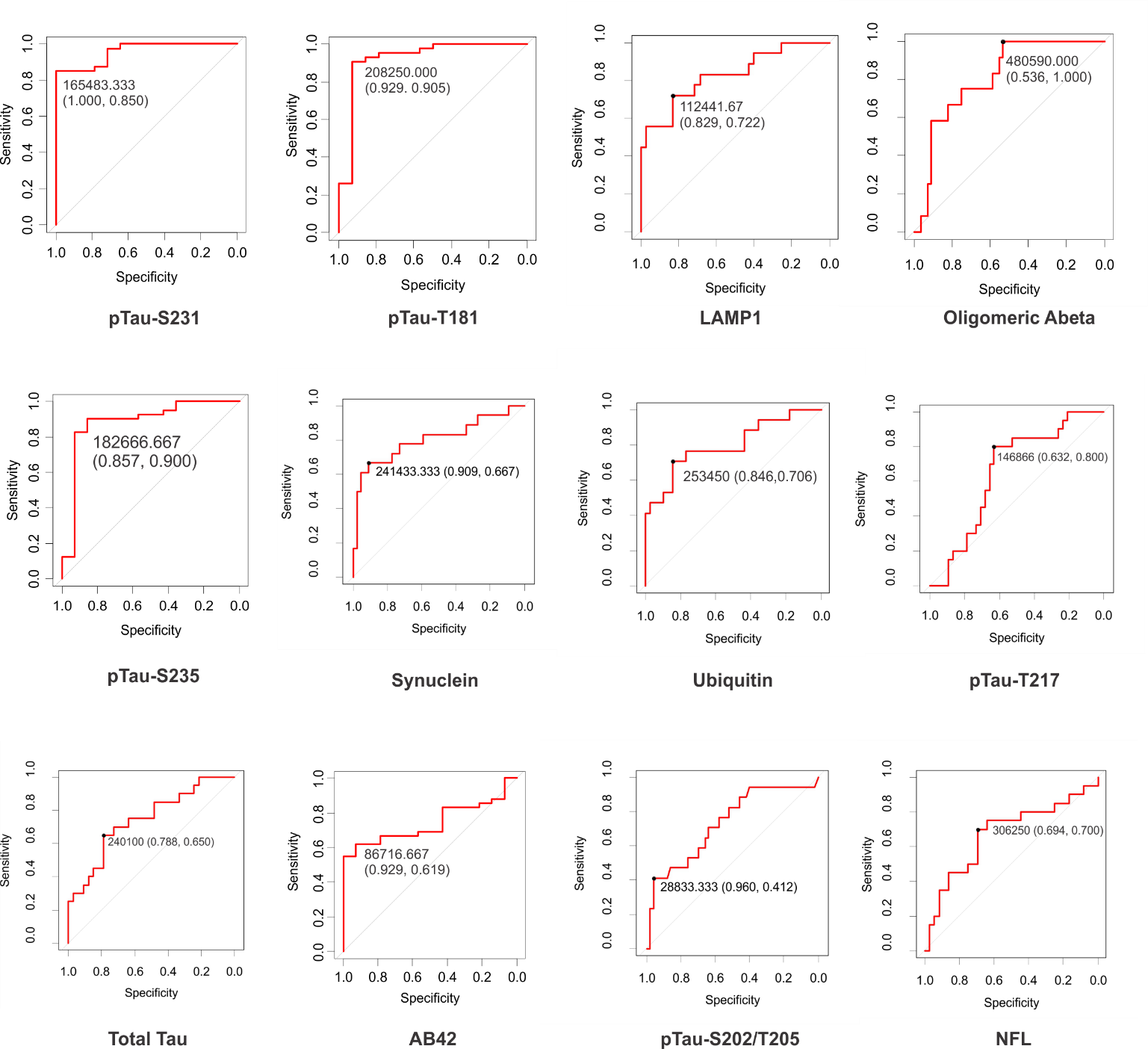


**Figure S4:** Combinations of individual labelled biomarkers that visually discriminate HC plasma samples from MCI or AD (mild vs. moderate) plasma samples.


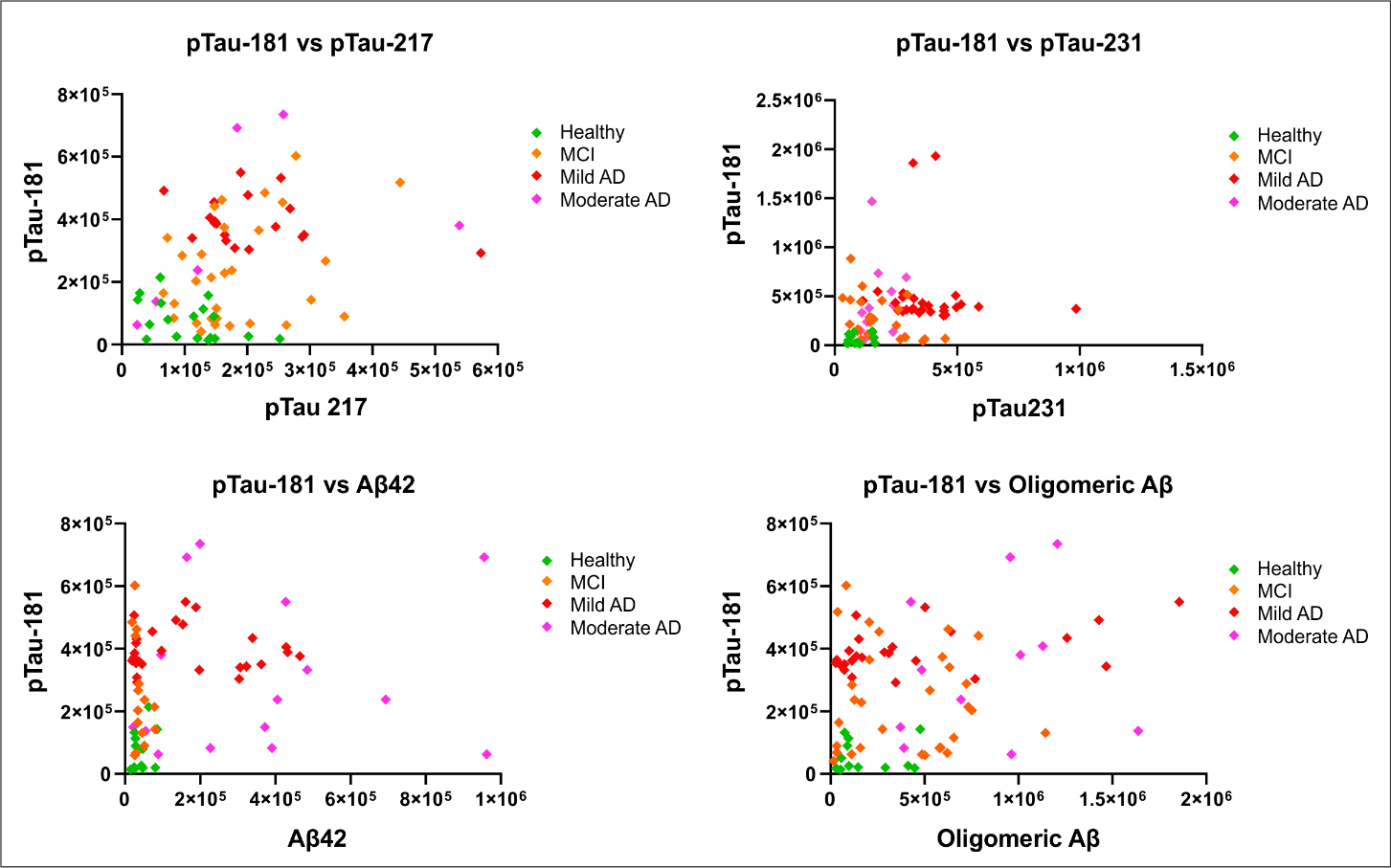


**Figure S5:** Western Blot and ELISA of markers on isolated EVs


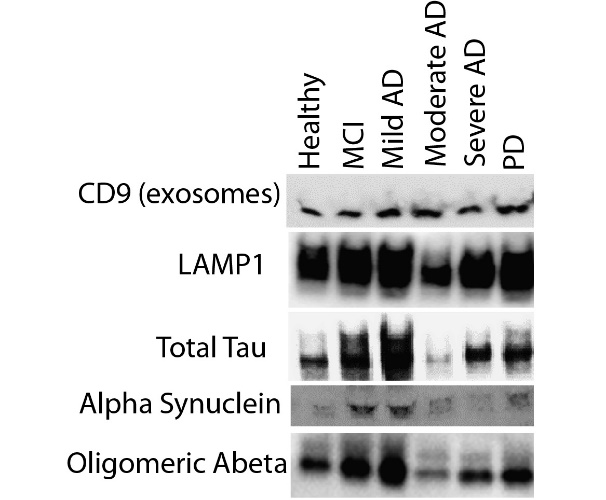

Supplement: Supplementary file 1 — Supporting information [file ALZ-20-6094-s001.docx]
